# Establishing severity levels for patient-reported measures of functional communication, participation, and perceived cognitive function for adults with acquired cognitive and language disorders

**DOI:** 10.1007/s11136-022-03337-2

**Published:** 2022-12-27

**Authors:** Matthew L. Cohen, Stacy M. Harnish, Alyssa M. Lanzi, Jennifer Brello, William D. Hula, David Victorson, Ratna Nandakumar, Pamela A. Kisala, David S. Tulsky

**Affiliations:** 1 Department of Communication Sciences and Disorders, University of Delaware, 100 Discovery Blvd 6th Floor, Newark, DE 19713 USA; 2 Center for Health Assessment Research and Translation, University of Delaware, Newark, DE 19713 USA; 3 Department of Speech and Hearing Science, The Ohio State University, Columbus, OH 43210 USA; 4 Geriatric Research, Education, and Clinical Center, VA Health Care System, and Department of Communication Sciences and Disorders, University of Pittsburgh, Pittsburgh, PA USA; 5 School of Medicine Department of Medical Social Science, Northwestern University, Chicago, IL 60611 USA; 6 University of Delaware School of Education, Newark, DE 19713 USA; 7 Department of Physical Therapy, University of Delaware, Newark, DE 19713 USA

**Keywords:** Patient-reported outcomes, Patient-reported outcome measures, Reference values, Reference standards, Cognitive impairments, Acquired communication disorders

## Abstract

**Purpose:**

To empirically assign severity levels (e.g., *mild*, *moderate*) to four relatively new patient-reported outcome measures (PROMs) for adults with acquired cognitive/language disorders. They include the Communicative Participation Item Bank, the Aphasia Communication Outcome Measure, and Neuro-QoL’s item banks of Cognitive Function (v2.0) and Ability to Participate in Social Roles and Activities (v1.0).

**Method:**

We conducted 17 focus groups that comprised 22 adults with an acquired cognitive/language disorder from stroke, Parkinson’s disease, or traumatic brain injury; 30 care partners of an adult with an acquired cognitive/language disorder; and 42 speech-language pathologists who had experience assessing/treating individuals with those and other cognitive/language disorders. In a small, moderated focus-group format, participants completed “PROM-bookmarking” procedures: They discussed hypothetical vignettes based on PROM item responses about people with cognitive/language disorders and had to reach consensus regarding whether their symptoms/function should be categorized as *within normal limits* or *mild*, *moderate*, or *severe* challenges.

**Results:**

There was generally good agreement among the stakeholder groups about how to classify vignettes, particularly when they reflected very high or low functioning. People with aphasia described a larger range of functional communication challenges as “mild” compared to other stakeholder types. Based on a consensus across groups, we present severity levels for specific score ranges for each PROM.

**Conclusion:**

Standardized, stakeholder-informed severity levels that aid interpretation of PROM scores can help clinicians and researchers derive better clinical meaning from those scores, for example, by identifying important clinical windows of opportunity and assessing when symptoms have returned to a “normal” range.

**Supplementary Information:**

The online version contains supplementary material available at 10.1007/s11136-022-03337-2.

Patient-reported outcome measures (PROMs) are standardized scales that assess health outcomes from the patient’s perspective [1], including health-related quality of life, functional status, symptoms, and health behaviors [2, 3]. In clinical settings, PROMs may be used to assist with screening and referral, diagnosis and estimating prognosis, monitoring symptoms over time, goal setting, monitoring treatment progress, and facilitating shared decision-making [4–9]. The use of PROMs in clinical practice supports the missions of evidence-based practice and person-centered care by quantifying health outcomes that matter to an individual client but that may not be fully observable to clinicians [7]. For example, people with cognitive/language disorders, their care partners, and speech-language pathologists (SLPs) often identify communicative *participation* (e.g., ordering at a restaurant) as an important therapeutic target in addition to or instead of communication *skills* (e.g., word-level repetition) [10–14]. However, participation-focused therapy requires assessment of communicative participation [10], which SLPs do less often [11]. Assessment of participation is inherently complex because “participation” measures need to reflect the convergence of communication skills, the environment, and personal perspectives to document whether the client is meeting their communication demands and preferences successfully and satisfactorily (Baylor & Darling-White, 2020, p.5). This makes communicative participation especially well-suited to be assessed by PROMs, and the Communicative Participation Item Bank (CPIB) was published by Baylor et al. (2013) to meet this need.

Within the last 20 years, several PROM systems have been developed using rigorous development standards and item-response theory (IRT). These include the Patient-Reported Outcomes Measurement Information System (PROMIS) [15], Neuro-QoL measurement system, Traumatic Brain Injury (TBI)-QOL measurement system [16], and Spinal Cord Injury (SCI)-QOL [17] measurement system. These systems were primarily designed for clinical research [18], but are increasingly being used for clinical purposes as well [4, 5, 8, 9, 19–21]. However, because they were not originally designed for clinical purposes, clinicians may question their applicability and usefulness [22]. For example, for many clinical purposes, clinicians find it useful to have simple guides for interpreting how “good” or “bad” the client’s score is [23] with terms such as *mild*, *moderate*, and *severe* [24]. Established score ranges can help facilitate and simplify screening, referring, and monitoring change, for example, by identifying important clinical windows of opportunity and assessing when symptoms have returned to a “normal” range [9, 23, 25]. Establishing and assigning descriptors to score ranges are typically not part of the psychometric development of new PROMs, so additional work is needed to make the measures optimally useful for clinicians.

One method for assigning severity levels to PROM score ranges is called bookmarking, a procedure originally used in the field of education whereby content experts set criteria for mastery of learned material [26]. Applied to PROMs, bookmarking engages stakeholders (e.g., patients, care partners, and clinicians) to determine how PROM scores should be interpreted [23, 24, 27–30]. This procedure involves groups of stakeholders reviewing hypothetical vignettes based on PROM scores about a person’s experience of a particular health construct. Vignettes are carefully written to correspond with actual T-score levels from a PROM. The stakeholder groups discuss and order the vignettes and then achieve consensus on where bookmarks should be placed between them to denote distinct severity levels such as normal, mild, moderate, or severe [6].

In the present paper, we describe some of the main findings of a study that aimed to set clinical cut points for a set of relatively new, IRT-based PROMs that might benefit clinical practice with adults with acquired cognitive/language disorders [6, 7, 31], particularly speech-language therapy. Specifically, we report the results of bookmarking groups that set clinical cut points for two measures of participation, the CPIB [32] and the Neuro-QoL Item Bank v1.0—Ability to Participate in Social Roles and Activities (NQ-SRA) [33, 34], a measure of functional communication, the Aphasia Communication Outcome Measure (ACOM) [35], and a measure of perceived cognitive function, the Neuro-QoL Item Bank v2.0—Cognitive Function (NQ-Cog) [33, 34]. These constructs have broad relevance to clinical practice with adults with acquired cognitive/language disorders and are well captured by PROMs.

## Methods

### Participants

This study aimed to develop cut points that were broadly applicable to adults with acquired cognitive/language conditions, based on input from different stakeholders and representative conditions. Stakeholders included patients, care partners, and SLPs. Conditions that caused cognitive/language challenges included traumatic brain injury (TBI), stroke, and Parkinson’s disease (PD)—some of the most common conditions that lead a person to seek cognitive/language treatment from an SLP. In line with previous PRO-Bookmarking studies [27, 29, 30, 36], we aimed to enroll a single group for each stakeholder and condition type (e.g., 1 TBI client group, 1 TBI care partner group, 1 stroke client group, 1 stroke care partner group, etc.). We ended up exceeding the planned enrollment for some groups but not all.

In the end, we conducted 17 focus groups—4 stroke client groups (*n* = 13), 4 stroke care partner groups (*n* = 19), 1 PD client group (*n* = 6), 1 PD care partner group (*n* = 7), 1 TBI client group (*n* = 3), and 1 TBI care partner group (*n* = 4). These groups approached the PRO-Bookmarking task based on shared lived experiences with their condition. We also enrolled 5 SLP groups (*n* = 42) that set bookmark locations that they thought were applicable to any adult client with a cognitive/language condition. Details of these participants and groups can be found in Table 1.

**Table 1:** Participants and groups

ID	Participants	Age: range (median)	Sex	Race	Ethnicity	Experience: range (median)	PROMs bookmarked
Clinicians						Years as SLP	
A	7 SLPs	30–62 (40)	7 w, 0 m	7 White	7 Non-Hispanic	6–34 (10)	1,2,3,4
B	10 SLPs	25–54, 3 did not report (33)	9 w, 1 m	9 White, 1 Black	10 Non-Hispanic	1–20 (6.5)	1,2,3,4
C	8 SLPs	27–40 (33.5)	8 w, 0 m	6 White, 1 Asian, 1 Other	8 Non-Hispanic	3.5–14 (9.5)	1,2,3,4
D	6 SLPs	26–65 (55.5)	6 w, 0 m;	6 White	6 Non-Hispanic	3–42 (22.5)	1,3,4
E	11 SLPs	27–67 (48)	11 w, 0 m	10 White, 1 Black	11 Non-Hispanic	3–30 (25)	1,2,4

Care partners						Years as care partner	
F	4 partners of PWA	60–76 (69)	3 w, 1 m	3 White, 1 Asian	1 Hispanic, 3 non-Hispanic	1–7.5 (1.5)	1,2,3,4
G	6 partners of PWA	35–69 (61.5)	6 w, 0 m	4 White, 2 Black	6 Non-Hispanic	2–10 (5.25)	1,2,3,4
H	4 partners of PWA	41–68, one did not report (62)	4 w, 0 m	4 White	4 Non-Hispanic	1–5.5 (3.25)	1,2,3,4
I	5 partners of PWA	48–75 (62)	4 w, 1 m	5 White	5 Non-Hispanic	1–18 (3)	1,2,3,4
J	7 partners of PwPD	55–76 (67)	6 w, 1 m	7 White	7 Non-Hispanic	2–15 (7)	1,3
K	4 partners of PwTBI	57–77 (64.5)	3 w, 1 m	4 White	4 Non-Hispanic	2–46 (14)	1,2,3

People with communication disorders						Years with condition	
L	6 PwPD	58–76 (68)	1 w, 5 m	6 White	6 Non-Hispanic	2–15 (8.5)	1,3
M	3 PWA	49–76 (62)	0 w, 3 m	2 White, 1 Black	3 Non-Hispanic	1.5–4.5 (3.5)	1
N	2 PWA	49–76 (62.5)	0 w, 2 m	2 White	2 Non-Hispanic	1.5–3.5 (2.5)	2,3,4
O	3 PWA	38–69 (53)	0 w, 3 m	2 White, 1 Black	3 Non-Hispanic	1.5–7 (4)	1,2,3,4
P	5 PWA	38–73 (55)	1 w, 4 m	4 White, 1 Black	5 Non-Hispanic	1–6 (4)	1,2,3,4
Q	3 PwTBI	30–77 (46)	0 w, 3 m	3 White	3 Non-Hispanic	3–23 (5)	1,2,3

SLP = speech-language pathologist, PWA = person with aphasia; PwPD = person with Parkinson’s disease; PwTBI = person with TBI. PROMs Bookmarked: 1 = Communicative Participation Item Bank, 2 = Neuro-QoL Cognition, 3 = Neuro-QoL Ability to Participate in Social Roles and Activities, 4 = Aphasia Communication Outcome Measure. Group IDs here do not match those presented in Cohen, Harnish et al. (2021). All demographic variables were self-identified by participants

The presence of an acquired cognitive/language condition was determined by clients and care partners endorsing one or more of the following challenges: Expressing oneself; other people understanding what one wants to say; understanding what other people say; reading or writing because of language difficulties (i.e., not because of visual or motor difficulties); thinking of the right word to say; moving one’s mouth, lips, or tongue to pronounce words; or thinking or memory. Participants with TBI or stroke were at least 6 months post-onset. Participants with these conditions were familiar to the research team by virtue of having participated in other research studies. They were invited to participate in this study because their cognitive/language challenges were thought to be representative of their condition but not too severe to preclude participation in the PRO-bookmarking tasks. For descriptive purposes, the severity of language impairment for people with aphasia was assessed with the Quick Aphasia Battery [37] or Comprehensive Aphasia Test [38] and was characterized by these performance-based measures as being mild or moderate (for more details, see [39]). People with PD or TBI produced Montreal Cognitive Assessment (MoCA) [40] total scores between 19–27 (median = 23.5), which are in the range of scores produced by people with mild neurocognitive disorder, overlapping with the upper end of scores produced by people with major neurocognitive disorder [40, 41].

Care partners were spouses of a person with a cognitive/language condition who had at least weekly contact with the person both before and after the onset of the condition. The inclusion criteria permitted other close relations, but the care partners in our sample were all spouses. SLPs were certified by the American Speech-Language-Hearing Association and had experience treating at least 50 adults with acquired cognitive/language conditions. All participants were at least 18 years old and could read and understand spoken English. All study procedures were approved by the Institutional Review Boards at the University of Delaware and The Ohio State University. Participants provided written informed consent, and participants with cognitive/language disorders were required to pass a series of yes/no comprehension questions to ensure their cognitive/linguistic capacity to provide informed consent. Participants were compensated for their time.

### Measures

#### Communicative Participation Item Bank (CPIB) [32]

The CPIB is a 46-item PROM that assesses communicative participation, defined as “taking part in life situations in which knowledge, information, ideas, or feelings are exchanged” [32, 42]. A 10-item short form is also available. It was initially calibrated on a sample of adults with multiple sclerosis, Parkinson’s disease, amyotrophic lateral sclerosis, and head and neck cancer [32], and was later found to have evidence for validity with people with aphasia as well [43]. Examples of items on the CPIB include “Does your condition interfere with getting your turn in a fast-moving conversation? and “Does your condition interfere with asking questions in a conversation?”.

#### Aphasia Communication Outcome Measure (ACOM) [35]

The ACOM consists of 59 items that assess post-stroke functional communication, defined as “the ability to engage in common, everyday behaviors, tasks, activities, and life situations that involve understanding and/or producing spoken, written, and/or non-verbal messages, signs, and symbols” (Doyle et al., 2008, p720 as cited in Hula et al., 2015). ACOM items assess spoken language expression and comprehension, reading, writing, and number use. Examples of ACOM items include “How effectively can you find the words you want to say during communication?” and "How effectively can you say the name of common objects (e.g., bed, lamp, pencil)?”.

#### Neuro-QoL Cognitive Function Item Bank (v2.0) (NQ-Cog) [33, 34]

The NQ-Cog consists of 28 items that assess “perceived difficulties in cognitive abilities (e.g., memory, attention, and decision-making) or in the application of such abilities to everyday tasks (e.g., planning, organizing, calculating, remembering, and learning)” [44]. Short forms are also available. Examples of NQ-Cog items include “How much difficulty do you currently have checking the accuracy of financial documents, (e.g., bills, checkbook, or bank statements)?” and “In the past 7 days, I had trouble keeping track of what I was doing if I was interrupted.”

#### Neuro-QoL Ability to Participate in Social Roles and Activities (v1.0) (NQ-SRA) [33, 34]

The NQ-SRA consists of 45 items that assess the “degree of involvement in one’s usual social roles, activities, and responsibilities; including work, family, friends, and leisure” [44]. Short forms are also available. As with the CPIB, it assesses aspects of participation but is not specific to communicative participation. Examples of NQ-SRA items include “In the past 7 days I have been able to do all of my regular activities with friends” and “In the past 7 days I have been able to keep up with my family responsibilities.”

### Bookmarking materials and procedures

Bookmarking is a process by which stakeholders (clients, care partners, and clinicians) help determine how PROM score ranges should be clinically interpreted [23, 24, 27–29, 45]. A previous publication describes how we have made the bookmarking materials and procedures more accessible to individuals with cognitive/language conditions [39]. These materials and procedures are described briefly below and in more detail in the Supplementary Material.

The first step was to create vignettes about hypothetical clients with acquired cognitive/language conditions based on PROM items and responses. For example, two CPIB items and item responses could be “José’s condition interferes quite a bit talking with people that he does not know, but only a little bit talking with people that he does know.” As also described in our previous publication [39] the selection of items followed Victorson’s guidelines [46]. Consistent with previous PRO-Bookmarking studies [28, 29], we developed about ten vignettes per item bank and each vignette contained five items/responses. Vignettes were written at 0.5 SD intervals for the CPIB, ACOM, and NQ-Cog, and every 0.25 SD intervals for the NQ-SRA. The reason that the vignettes for the NQ-SRA were closer together is that there was less variability in most-likely responses in items at T-score levels < 35 and > 50, so we distributed the ten vignettes across a smaller range of scores to permit more precise classification. Each vignette was assigned an arbitrary surname (e.g., “Mr. Garcia”), and surnames represented the most common ancestries in the USA.

**Fig. 1:**
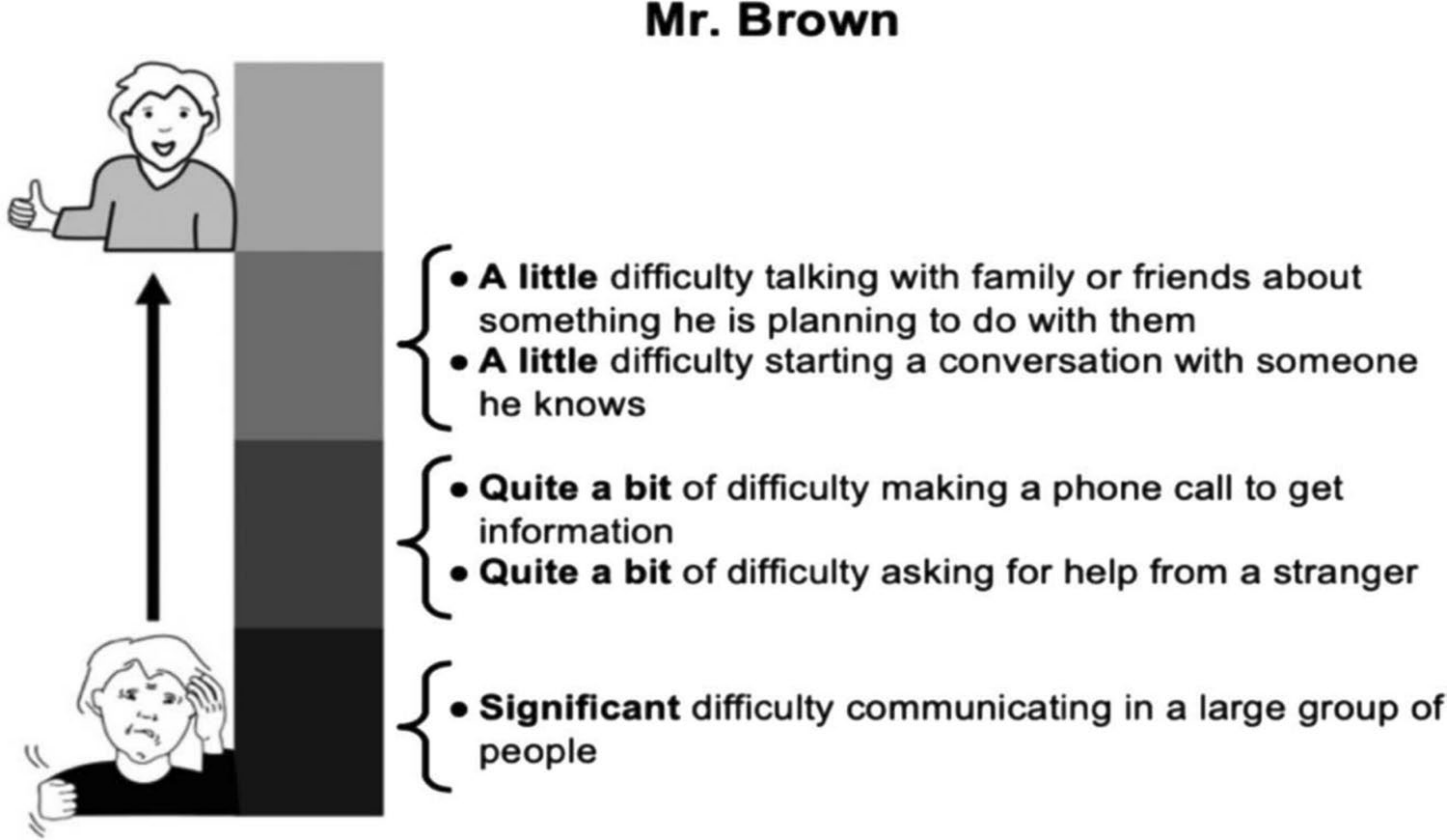
Example of a vignette card from Cohen et al., (2021). This card depicts “Mr. Brown” who represents a CPIB T score of 40. As further described by Cohen et al. (2021), participants had 8–10 cards per PROM and discussed where bookmarks should be placed between adjacent cards that were in order and represented different scores. Reprinted from Cohen et al. (2021) with permission from the publisher

The second step was to put those vignettes on physical cards (8.5″ × 5.5″ cardstock) that could be viewed, sorted, and manipulated by participants. To be maximally accessible to individuals with communication disorders, the vignettes were displayed in large font with bullet points and with a graphic to indicate whether the items/responses described a high or low level of the trait being assessed [39]. Figure 1 shows an example of a CPIB vignette card. Representative examples of vignettes from every item bank are freely available as supplementary material to our previous publication [39].

**Fig. 2:**
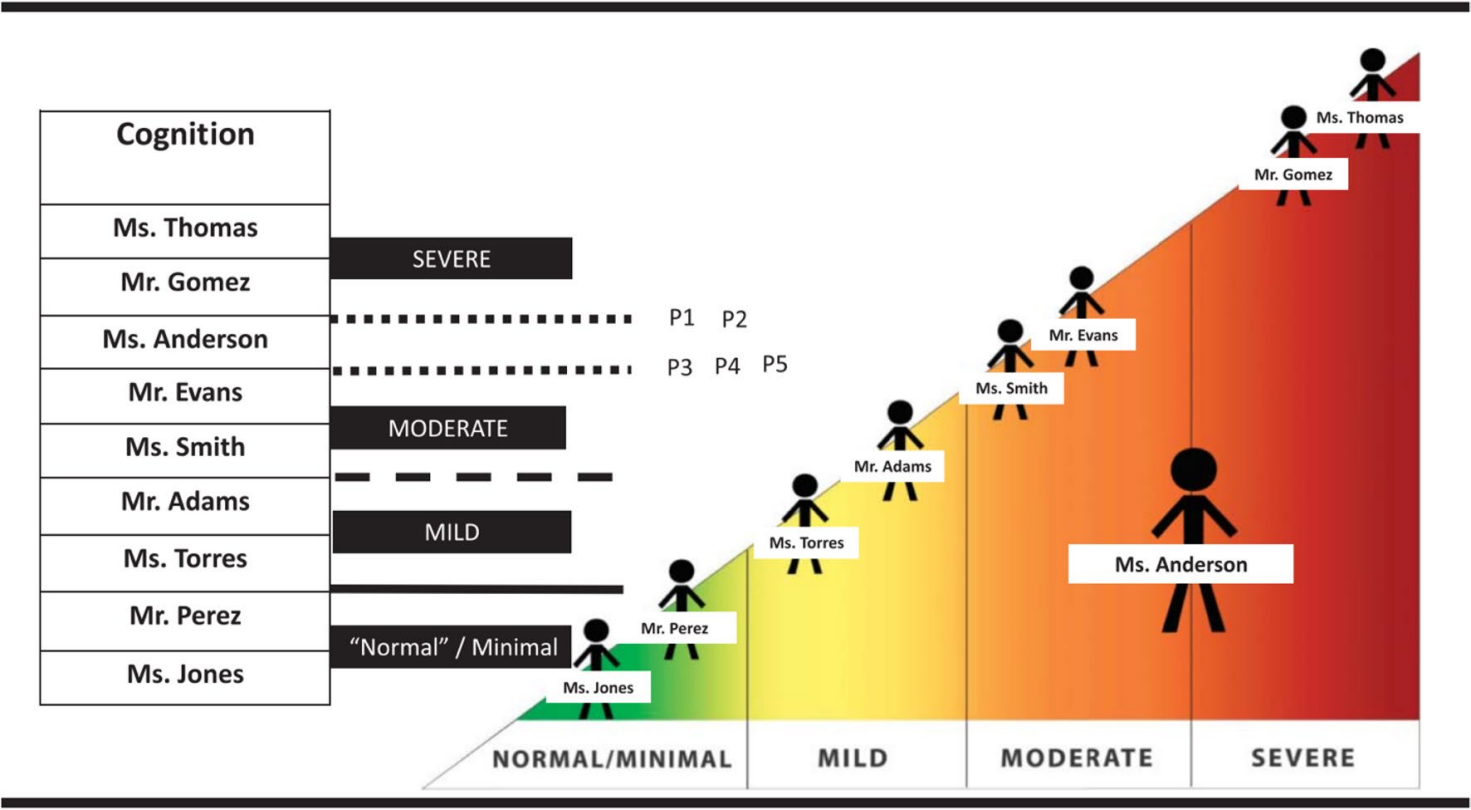
Powerpoint display that was developed from feedback from stakeholders and used to graphically support the PRO-bookmarking task. As also described in Cohen et al. (2021), Fig. 2 depicts a scenario in which participants have agreed on all bookmark locations except the boundary between moderate and severe. It is being discussed whether “Ms. Anderson” should be classified as moderate or severe. Graphically, Ms. Anderson is the largest figure, indicating that she is the vignette under discussion. Previously classified vignettes are smaller, in psychometric order, and within the category boundary to which participants have assigned them previously in the discussion. Participants 1 and 2 think Ms. Anderson should be classified as moderate (that is, the boundary for severe is between her and Mr. Gomez); Participants 3, 4, and 5 think she should be classified as severe (that, the boundary for severe is between her and Mr. Evans). As participants are persuaded and change their position, the moderator uses multimodal support to make the consensus-building process. Reprinted from Cohen et al., 2021 with permission from the publisher

The third step was to conduct bookmarking groups separately for each stakeholder and condition type: Adults with post-stroke aphasia, TBI, or PD (6 groups); care partners of people with aphasia, TBI, or PD (6 groups); and SLPs (5 groups) (Table 1). We adapted the bookmarking procedure to be accessible to people with cognitive/language conditions by breaking down tasks into small components, displaying activities graphically when possible, and using moderator(s) who were able to give impromptu communication support as needed because of their clinical training as a psychologist (authors M.L. and D.V.) or speech-language pathologist (authors A.L., J.B., and S.H.) [39]. After providing informed consent and completing introductions and practice examples, participants were introduced to a PROM and what the response options look like to respondents. They were then given the vignette cards associated with that PROM and asked to spend some time becoming familiar with them. Participants would then place “bookmarks” between adjacent vignettes that they felt represented a boundary between people whose challenges they would classify as *within normal limits,* or *mild*, *moderate*, or *severe* challenges. Group members sometimes disagreed in their initial placement of bookmarks, and it was the job of the moderator(s) to facilitate a discussion that led to a consensus regarding why one vignette person’s challenges were *mild* but the adjacent person’s was *moderate*. Figure 2 depicts a visual communication aid used during this discussion. In the end, each group achieved consensus about the location of bookmarks for the item banks they were able to complete. As also discussed by Cohen, Harnish et al., [39] not every group bookmarked every item bank due to time, appropriateness to the condition, and other constraints. For example, TBI and PD groups did not bookmark the ACOM because it is specific to post-stroke aphasia. The PROMs were not bookmarked in a fully counterbalanced order. Because it was initially unclear how long the task would take with each stakeholder group, item banks were bookmarked in order of their importance to the aims of the grant that funded this investigation: first the CPIB, then the ACOM (for aphasia stakeholders only), then NQ-Cog or NQ-SRA followed by the other. Table 1 shows which groups bookmarked which item bank.

### Placement of consensus cut points

The authors of this paper served as an adjudicating expert panel to interpret and synthesize the results of multiple bookmarking groups into a final set of cut points. When the groups largely agreed on the classification of adjacent vignettes, for example, T45 indicates *mild* challenges and T40 indicates moderate challenges, the consensus bookmark location was placed between them at T42.5. However, when the groups were split about how to classify a vignette, e.g., whether the vignette at T35 should be called *moderate* or *severe*, the consensus bookmark location was placed at that level such that any score above 35 is *moderate* and any score below 35 is *severe*.

## Results

### Cut points

**Fig. 3:**
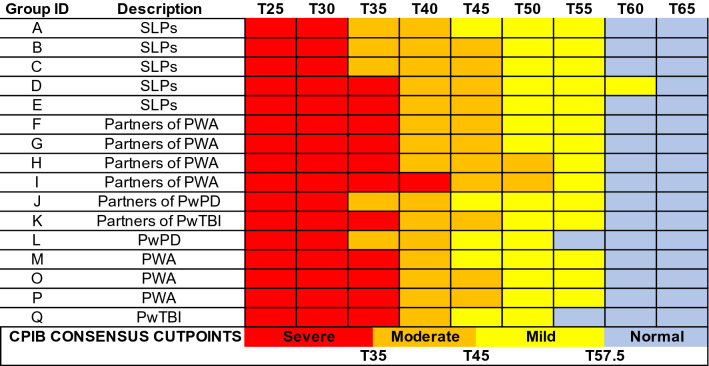
Cut points for the Communicative Participation Item Bank. CPIB = Communicative Participation Item Bank. Vignettes are shown by their associated T score (*M* = 50, SD = 10), where a T score equal to 50 matches the mean of that measure’s reference sample. For the CPIB, that reference sample is 701 individuals with multiple sclerosis, Parkinson’s disease, amyotrophic lateral sclerosis, and head and neck cancer. Each colored cell represents how each group classified each vignette (e.g., the T25 vignette, the T30 vignette)—within normal limits (blue), mild (yellow), moderate (orange), or severe (red). The synthesized cut points, determined by the adjudicating expert panel, are at the bottom

**Fig. 4:**
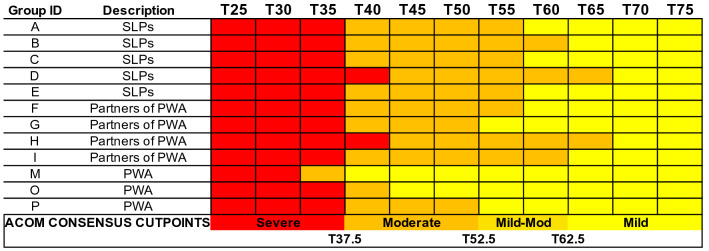
Cut points for the Aphasia Communication Outcome Measure. Vignettes are shown by their associated T score (M = 50, SD = 10), where a T score equal to 50 matches the mean of that measure’s reference sample. For the ACOM, that reference sample is 329 PWA. Because every participant in the reference sample had aphasia, there is no “normal” range of functional communication. Each colored cell represents how each group classified each vignette (e.g., the T25 vignette, the T30 vignette)—within normal limits (blue), mild (yellow), moderate (orange), or severe (red). The synthesized cut points, determined by the adjudicating expert panel, are at the bottom

**Fig. 5:**
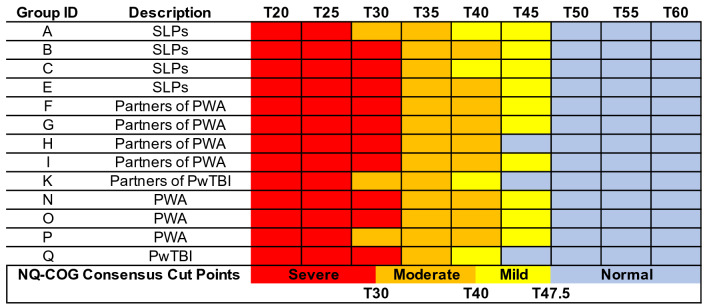
Cut points for the Neuro-QoL Item Bank v2.0—Cognitive Function. NQ-Cog = Neuro-QoL Item Bank v2.0—Cognitive Function. Vignettes are shown by their associated T score (*M* = 50, SD = 10), where a T score equal to 50 matches the mean of that measure’s reference sample. For the NQ-Cog, that reference sample matches a U.S. census-matched general population [53]. Each colored cell represents how each group classified each vignette (e.g., the T25 vignette, the T30 vignette)—within normal limits (blue), mild (yellow), moderate (orange), or severe (red). The synthesized cut points, determined by the adjudicating expert panel, are at the bottom

**Fig. 6:**
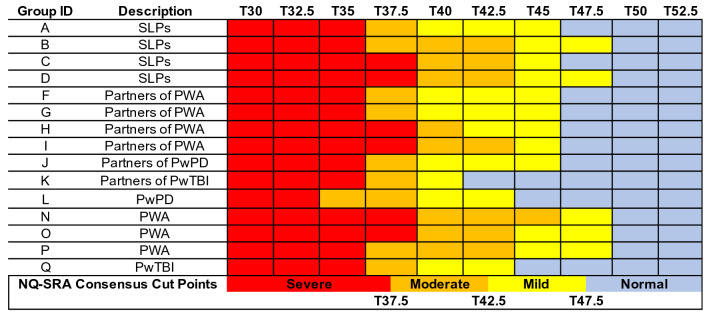
Cut points for the Neuro-QoL Item Bank v1.0—Ability to Participate in Social Roles and Activities. NQ-SRA = Neuro-QoL Item Bank v1.0—Ability to Participate in Social Roles and Activities. Vignettes are shown by their associated T score (*M* = 50, SD = 10), where a T score equal to 50 matches the mean of that measure’s reference sample. For the NQ-SRA, that reference sample matches a U.S. census-matched general population [53]. Each colored cell represents how each group classified each vignette (e.g., the T25 vignette, the T30 vignette)—within normal limits (blue), mild (yellow), moderate (orange), or severe (red). The synthesized cut points, determined by the adjudicating expert panel, are at the bottom

Figures 3, 4, 5, and 6 visually depict how each PRO-bookmarking group classified vignettes and the consensus cut points. The group IDs in Figs. 3, 4, 5, and 6 match the group IDs in Table 1 so the reader can see details about group characteristics. For the CPIB (Fig. 3), scores above T57.5 were found to represent “minimal problems” or a “normal” experience of communicative participation. Scores between T57.5 and T45 represent *mild* challenges, scores between T45 and T35 represent “moderate” challenges, and scores below T35 represent “severe” challenges.

For the ACOM (Fig. 4), the consensus cut points were set as follows. Scores above T62.5 represent “mild” aphasia, scores between T62.5 and T52.5 represent “mild-to-moderate” aphasia, scores between T52.5 and T37.5 represent “moderate” aphasia, and scores below T37.5 represent “severe” aphasia. Reaching consensus on these cut points required more consideration than did the other PROMs. PWA extended the “mild” range of challenges further than other stakeholder groups. Because of this, the most contested range (T-scores 62.5—52.5) was called “mild-to-moderate.” Because every participant in the reference sample had aphasia, there is no “normal” range of functional communication on the ACOM.

For the NQ-Cog, scores above T47.5 represent “minimal problems” or a “normal” experience of cognitive errors. Scores between T47.5 and T40 represent “mild” challenges, scores between T40 and T30 represent “moderate” challenges, and scores below T30 represent “severe” challenges. When Group H (partners of PWA) bookmarked the NQ-Cog they chose not to classify any vignette as “mild” (Fig. 5). This was the only example of a group deciding that a descriptor did not apply to any vignette. When we as the study team served as an adjudicating expert panel to set consensus bookmark locations, we decided that because all other groups perceived a score range as “mild,” the category was justified in the consensus decision.

For the NQ-SRA, scores above T47.5 represent “minimal problems” or a “normal” experience of participation in social roles and activities. Scores between T47.5 and T42.5 represent “mild” challenges,” scores between T42.5 and T37.5 represent “moderate” challenges, and scores below T37.5 represent “severe” challenges (Fig. 6). For all item banks, scores that fall exactly on the cut point could be described as “moderate-severe”, “mild-moderate,” or “borderline.”

## Discussion

PROMs are making increasingly important contributions to evidence-based practice and person-centered care by measuring important outcomes that are otherwise difficult to quantify [7]. Several relatively new PROMs have been developed that are intended for or highly relevant for adults with acquired cognitive/language disorders, and both clients and clinicians have much to gain from PRO-informed clinical practice [7, 31, 47]. Previous studies have indicated that clinicians find it helpful to have descriptors such as mild, moderate, and severe to describe PROM score ranges [24], so the purpose of this study was to map those descriptors onto score ranges on these PROMs. In a previous publication [39], we reported how we adapted the bookmarking procedure to be accessible to individuals with acquired cognitive/ language impairments. Here, we report the results from bookmarking groups that set cut points for the CPIB, ACOM, NQ-Cog, and NQ-SRA.

The healthmeasures.net website, which hosts PROMIS, Neuro-QoL, and other measurement systems, has rule-of-thumb guidance for interpretation of the NQ-SRA and NQ-Cog based on the score distributions from calibration testing data [15, 48]. The guideline is that for measures that reference a general population sample, T-scores above T45 are considered “within normal limits,” scores between T45-40 are mild, scores between T40 and T30 are moderate, and scores below T30 are severe. To our knowledge, this is the first report of PRO-bookmarking-derived clinical cut scores for the NQ-SRA and the second study to report bookmarking-derived scores for the NQ-Cog.

The first study that reported cut points for the NQ-Cog, Rothrock et al. [24], conducted a single bookmarking group with people with cancer and a single bookmarking group with oncologists. Overall, the NQ-Cog cut points set by our stakeholder groups are grossly congruent with (i.e., ≤ 0.5SD different from) the stakeholder groups reported by Rothrock et al. and with the guidelines of healthmeasures.net. Our groups’ cut points for the NQ-SRA are grossly congruent with the healthmeasures.net guidelines for the mild and moderate thresholds, but different for the threshold of severe; whereas healthmeasures.net indicates that the severe range is for scores < T30, our consensus cut points for that measure is for scores < T37.5. This means that our group assigned the descriptor “severe” to a range of scores (T-scores from 30.0 up to 37.5) that healthmeasures.net would call moderate. The reason for this discrepancy is not clear, except that our cut point was based on bookmarking (i.e., the impression of stakeholders) rather than based on score distributions. Ultimately, however, clinically meaningful interpretation of PROM scores is most valid when interpretation guides are specific to the population completing the PROM [49]; what is considered a “mild” cognitive limitation for a person with cancer might be different than for a person with an acquired cognitive/language disorder.

This is the first study to report descriptors for CPIB score ranges. It is notable that the “mild” range begins at T57.5, a much higher T-score threshold than the NQ-Cog or NQ-SRA item banks. This was most-likely related to the composition of the reference sample. Whereas the reference samples for the NQ-Cog and NQ-SRA mirror the general population [15, 48], where T50 indicates a truly typical experience [50], the reference sample for the CPIB was a sample of people with communication disorders. This means that *T* = 50 indicates a typical experience of communicative participation *for people with a communication disorder*; thus, it is not surprising that our stakeholders determined that the “normal” range is for scores notably higher than T50. This highlights the importance of PRO-bookmarking. Without clear descriptions of score ranges, it could be difficult for a clinician to know how to interpret a CPIB score of, say, T55. That score is 0.5 SD above the mean, indicating better-than-average participation, but would still be classified by most of our stakeholder groups as indicating mild restrictions to communicative participation.

Previous bookmarking studies have reported mixed consistency between stakeholder groups, perhaps related to the types of stakeholders, the constructs being assessed, and the number of groups that were conducted. For the CPIB. NQ-Cog, and NQ-SRA, there was good consensus among our groups and so the consensus cut points were relatively faithful to the individual group cut points without needing to reconcile major differences. There was less consistency between PWA groups and other stakeholders who bookmarked the ACOM, and the discrepancies were in the same direction as previous bookmarking reports; patient groups set higher thresholds (i.e., “tolerate” more symptoms) than clinician or care partner groups [24, 27, 29]. For example, Cook et al. [27] reported that people with multiple sclerosis agreed with clinicians on the cut points for PROMs of mobility and sleep disturbance, but set higher thresholds for PROMs that assessed fine motor function and fatigue. One possible explanation for this finding is that people with the condition acclimate to its symptoms (i.e., recalibrate what constitutes a “mild problem”) [27, 29]. Curiously, however, PWA (and other patient groups) did not place bookmarks in that way for any other PROM/construct. In fact, on the NQ-SRA they had a *lower* threshold (i.e., tolerated fewer symptoms) of restricted participation in social roles and activities than did care partners and half of the SLP groups, labeling T47.5 as “mild” whereas most others called it “normal.”

Despite these occasional discrepancies, there is generally good agreement among groups. One strength of this study is the number of groups that were conducted. Whereas previous PRO-bookmarking studies generally conducted a single group for each condition or stakeholder type [27, 29, 30, 36], we conducted multiple groups. This helps improve confidence in the consensus cut points. Still, it is difficult to know when saturation is reached—the point at which the addition of new data fails to change the consensus bookmark locations. The consensus cut points based on 12–16 focus groups are likely more stable than the cut points of individual groups, so interpretation of subgroup (e.g., PWA-only) cut points requires caution. Our SLP and aphasia stakeholder groups had the largest sample sizes, so those perspectives are likely the most stable (i.e., likely to be saturated). Although the cut points placed by other stakeholder types largely converged with those set by SLPs and aphasia stakeholders, it is possible that the addition of more data would reveal differences. It would be useful for future research to test these bookmark locations with more perspectives from TBI and PD stakeholders, as well as the perspectives of patients with other neurogenic communication disorders. We also cannot rule out the possibility that cut points were influenced by the items included in the vignettes, the order in which the PROMs were bookmarked, or the members that comprised the groups.

The results presented here are intended to help make these four PROMs more interpretable for clinicians and researchers who serve adults with cognitive/language disorders, improving their ability to quantify aspects of quality of life, especially those aspects that are difficult to quantify otherwise [7]. However, we would caution against over-reliance on these cut points alone for major clinical decision-making such as whether a disorder is present or not. It is important to keep in mind that these cut points are not meant to diagnose a clinical condition, they do not necessarily apply to other causes of disorders that are not represented here, and our vignettes were limited to 0.25 or 0.5 SD intervals, limiting the precision of the cut points. Instead, the score ranges presented here can help clinicians determine roughly “how good or bad” a particular score is based on the average client with that level of symptoms/function. However, the descriptor may not apply to a particular client for whom participation, for example, is more of a priority than for the average client. Other important interpretation tools for PROMs include the minimal detectable change and minimally important difference values, which help determine “how much better or worse” a particular score is compared to a previous score [39, 49, 51, 52]. PROMs can serve as very useful data to include in a clinician’s delivery of evidence-based practice, but they are most valid and useful when interpreted alongside other assessment data (e.g., performance-based and clinician-rated assessments) [7].

## Supplementary Information

Below is the link to the electronic supplementary material.

Supplementary file1 (DOCX 16 kb)

## Data Availability

The datasets generated and/or analyzed during the current study are not publicly available because additional publications are planned but are available from the corresponding author upon reasonable request.
