# Supplementary material for: Establishing severity levels for patient-reported measures of functional communication, participation, and perceived cognitive function for adults with acquired cognitive and language disorders
Source: Qual Life Res. 2022 Dec 27;32(6):1659–70. doi: 10.1007/s11136-022-03337-2 (PMC10172211; doi:10.1007/s11136-022-03337-2)
Supplement: Supplementary file 1 — Supplementary file1 (DOCX 16 kb) [file 11136_2022_3337_MOESM1_ESM.docx]

**Supplementary Information**

**Bookmarking Materials**

The first step was to create vignettes about hypothetical clients with acquired cognitive/language conditions based on PROM items and responses. For example, two CPIB items and item responses could be “José’s condition interferes quite a bit talking with people that he does not know, but only a little bit talking with people that he does know.” As also described in our previous publication [39] the selection of items followed Victorson’s guidelines [46]. First, as much as possible, we selected items to provide varied responses at the trait level described by the vignette, in order to avoid, e.g., a constant response of “never” to each item. Second, if items had to be repeated across vignettes, we tried to repeat them consistently. Third, we tried to avoid repeating items in consecutive vignettes so that participants had to consider the underlying trait rather than simply compare different observed responses to the same item. Finally, we tried to create vignettes that were clinically realistic—for example, by avoiding items and responses that seemed to contradict one another. The arbitrary names of people described by the vignettes were drawn from a list of the most common family surnames in the United States. Each surname was used in only one vignette. Pronoun gender was alternated between male and female across consecutive vignettes. The vignettes were printed on half a page (8.5′′×5.5′′) of color cardstock with a different color paper for each item bank. Figure 1 shows an example of a CPIB vignette card. Representative examples of vignettes from every item bank are freely available as supplementary material to our previous publication [39]. The item responses associated with each T-score level were computed based on the published IRT parameters of each item bank and using the R program published by Morgan et al. [30]. This step was completed in line with previous bookmarking reports [27–30] using the published unidimensional parameters of the CPIB, NQ-Cog, and NQ-SRA. However, a few additional steps were needed for the ACOM.

ACOM items displayed adequate fit to both unidimensional and bifactor measurement models [35]. We were not sure which model would be the most appropriate to use for this step, so we took an agnostic approach by computing most-likely responses two ways, once with the general factor of the bifactor model and again with the unidimensional parameters. Most-likely responses were 96% identical, and we simply avoided selecting those 4% of items and T-score levels where most-likely responses differed. ACOM items also represent different content areas of functional communication, including spoken language expression and comprehension, reading, writing, and number use. Pilot testing with SLPs suggested that it was difficult to compare the relative severities of challenges in spoken and written language, so we only selected items that assessed spoken language expression and comprehension. These items were still able to represent the full range of ACOM scores.
